# Effects of Four-Week Rehabilitation Program on Hemostasis Disorders in Patients with Spinal Cord Injury

**DOI:** 10.3390/jcm9061836

**Published:** 2020-06-12

**Authors:** Magdalena Mackiewicz-Milewska, Małgorzata Cisowska-Adamiak, Danuta Rość, Iwona Głowacka-Mrotek, Iwona Świątkiewicz

**Affiliations:** 1 Department of Rehabilitation, Nicolaus Copernicus University in Toruń, Collegium Medicum in Bydgoszcz, 85-094 Bydgoszcz, Poland; magmami@onet.eu (M.M.-M.); malgorzata.cisowska@cm.umk.pl (M.C.-A.); iwona.glowacka@cm.umk.pl (I.G.-M.); 2 Department of Pathophysiology, Nicolaus Copernicus University in Toruń, Collegium Medicum in Bydgoszcz, 85-094 Bydgoszcz, Poland; d.rosc@cm.umk.pl; 3 Department of Cardiology and Internal Medicine, Nicolaus Copernicus University in Toruń, Collegium Medicum in Bydgoszcz, 85-094 Bydgoszcz, Poland; 4 Division of Cardiovascular Medicine, University of California San Diego, La Jolla, CA 92037, USA

**Keywords:** spinal cord injury, rehabilitation, exercises, hemostasis, venous thrombosis

## Abstract

Background: Patients with spinal cord injury (SCI) exhibit hemostasis disorders. This study aims at assessing the effects of a 4-week rehabilitation program on hemostasis disorders in patients with SCI. Methods: Seventy-eight in-patients undergoing a 4-week rehabilitation were divided into three groups based on time elapsed since SCI: I (3 weeks–3 months), II (3–6 months), and III (>6 months). Tissue factor (TF), tissue factor pathway inhibitor (TFPI), thrombin–antithrombin complex (TAT) and D-dimer levels, antithrombin activity (AT), and platelet count (PLT) were measured on admission and after rehabilitation. Results: Rehabilitation resulted in an increase in TF in group III (*p* < 0.050), and decrease in TFPI (*p* < 0.022) and PLT (*p* < 0.042) in group II as well as AT in group I (*p* < 0.009). Compared to control group without SCI, TF, TFPI, and TAT were significantly higher in all SCI groups both before and after rehabilitation. All SCI groups had elevated D-dimer, which decreased after rehabilitation in the whole study group (*p* < 0.001) and group I (*p* < 0.001). Conclusion: No decrease in activation of TF-dependent coagulation was observed after a 4-week rehabilitation regardless of time elapsed since SCI. However, D-dimer levels decreased significantly, which may indicate reduction of high fibrinolytic potential, especially when rehabilitation was done <3 months after SCI.

## 1. Introduction

Venous thromboembolism (VTE) is a frequent complication in patients after spinal cord injury (SCI). Deep vein thrombosis (DVT) occurs in 5.4–27% and pulmonary embolism (PE) in 4–5.2% of patients, mainly in the acute period, although these complications have also been described in the chronic phase after SCI [1,2,3,4,5,6,7]. The risk of VTE is greatest (up to ~86% of all VTE events) in the first 3 months after injury [1,7]. The occurrence of VTE events drops significantly to about 9% between 3 and 6 months after injury and may still be observed at lower frequency (~5%) in long-term follow-up beyond 6 months [7]. Furthermore, our previous findings indicate that for patients beyond 3 months after SCI and diagnosed with DVT, 80% of DVT was detected during the period up to 6 months after injury [6]. The high risk of thromboembolic complications in SCI patients is associated with several factors including venous stasis resulting from immobilization, paresis or paralysis, decreased muscle contractibility, previous VTE, and infections [1,7,8]. In addition, patients with SCI exhibit hemostasis disorders, which can contribute to the VTE [6,9,10].

Rehabilitation of patients after SCI in the subacute or chronic phase involves regular physical activity of moderate intensity. The effect of a systematic moderate-intensity rehabilitation on hemostasis in the SCI patients has not been definitely assessed. Based on the results of previous studies that were conducted in other populations, it has been proven that exercise, especially of high intensity, has an impact on coagulation and fibrinolysis and may be responsible for the sudden death of persons participating in a marathon or triathlon [11,12]. On the other hand, it is widely known that regular, moderate exercise exerts a positive effect on the cardiovascular system [13].

In clinical practice, D-dimer concentration, platelet (PLT) count, and antithrombin (AT) activity are the most commonly measured parameters in patients with suspected VTE. Tissue factor (TF), tissue factor pathway inhibitor (TFPI) and thrombin–antithrombin (TAT) complex levels are rarely assessed in routine clinical practice. TF, previously known as thromboplastin, is a protein that initiates coagulation through the so-called extrinsic coagulation pathway [14,15,16]. It serves as a receptor for factor VII with which it forms the TF/VII complex, resulting in thrombin formation as well as deposition of fibrin and platelets in the process of clot formation [16,17,18,19]. TPFI is an endogenous physiological inhibitor of the TF/VII complex. Its anticoagulant properties are involved only in the coagulation pathway initiated by TF [20,21]. TAT complex is an indicator of prothrombin to thrombin activation and is one of the principal markers of blood hypercoagulability [22].

The objective of this research was to assess whether a four-week systematic rehabilitation involving moderate-intensity exercises affects selected parameters of coagulation and fibrinolysis in patients with SCI. To the best of our knowledge, our study is the first to investigate these interactions in such a specific population of patients. We measured the concentration of TAT complex, AT activity, D-dimer levels, PLT count, and extrinsic coagulation pathway factors, such as TF and TFPI levels. Our hypothesis was that regular, moderate exercise can reduce the activation of coagulation and fibrinolysis factors and, thus, may mitigate thromboembolic complications in the SCI patients.

## 2. Material and Methods

### 2.1. Study Design

This prospective study included patients with SCI who were hospitalized at the Department of Rehabilitation, the University Hospital No. 1 in Bydgoszcz, Poland, from 2011 to 2017. Every patient with SCI admitted to the Department of Rehabilitation in the period from 2011 to 2017 who agreed to participate was included in the study. Based on previous studies on the time course of the occurrence of VTE events post-SCI [1,6,7] as briefly summarized in Section 1, the patients were divided into the following three groups according to time elapsed since injury: group I (>3 weeks to 3 months, subacute phase), group II (>3 months to 6 months, early chronic phase), and group III (>6 months, late chronic phase). The study was approved by the local bioethical committee (KB 295/2011, 5 May 2011) and all patients signed an informed consent form.

All patients underwent clinical examination for assessment of paresis and symptoms of DVT such as edema, increased temperature, and erythema of the upper and lower limbs. They were also subjected to Doppler ultrasound examination of the lower-limb venous system and were graded according to the American Spinal Injury Association Impairment Scale (AIS). The occurrence of post-traumatic complications, such as decubitus ulcers, heterotopic ossifications, or urinary tract infection was also recorded (Table 1). PLT count, TF, TFPI, TAT complex and D-dimer plasma levels, and AT activity were measured in all patients at the time of admission and after four weeks of rehabilitation. Because normal reference ranges for TF, TFPI and TAT plasma concentrations were not available, their levels were also assessed in a control group of 42 individuals without SCI.

All patients underwent a four-week course of rehabilitation program, which included about two hours of moderate exercise a day with verticalization, wheelchair adaptation if necessary, passive and active exercises of the upper and lower limbs, resistance training, learning to move in a wheelchair, self-service, or learning to walk. The intensity of exercise was estimated as moderate but the maximal oxygen consumption (VO_2_ max) parameter that qualifies exercise as low, moderate, or high intensity was not assessed.

The plasma concentrations of TF, TFPI, and TAT complex were determined by enzyme-linked immunosorbent assay (ELISA) using specific IMUBIND^®^ kits (Sekisui Diagnostics). Plasma levels of D-dimer were determined by INNOVANCE^®^ D-Dimer (Siemens Healthcare Diagnostic Products GmbH), which is a particle-enhanced immunoturbidimetric assay for the quantitative determination of cross-linked fibrin degradation products (D-dimers). AT activity was measured by a chromogenic test, INNOVANCE Antithrombin (Siemens Healthcare). Platelets were counted with a Sysmex hematology analyzer.

There was no control group of patients with SCI who did not receive rehabilitation after injury due to the difficulty in accessing such patients, especially in the subacute stage up to three months after injury. If patients are not receiving rehabilitation during this period, it is usually because of severe complications of the trauma that preclude rehabilitation.

### 2.2. Statistical Analysis

Statistical analyses were conducted using Statistica 13.1 software (StatSoft Europe GmbH, Hamburg, Germany). Analyzed parameters were characterized by non-normal distribution. Hence, the non-parametric tests, Mann–Whitney U and Wilcoxon signed-rank, were used to evaluate the differences between groups for hemostasis parameters. For demographic and clinical characteristics, the differences between the groups were analyzed using the Kruskal–Wallis and post hoc Fisher’s Least Significant Difference tests. A probability level of *p* < 0.05 was considered statistically significant. Median (Me) and quartiles (lower Q25 and upper Q75) were used to describe the variables.

## 3. Results

Initially, 88 patients with SCI were enrolled in the study, but 10 patients discontinued rehabilitation prematurely, so 78 patients were included in the final analyses. Reasons for not completing the rehabilitation program included transfer to another hospital or a rehabilitation time of less than four weeks. The final group comprised 66 men (84.6% of total) and 12 women (15.4%). The average age of the patients in the study group was 37.7 years (±15.7 years); the women were older (42 ± 20.8 years) compared to men (37 ± 14.6 years). The minimum age was the same in the women and the men (18 years), while the maximum age varied; it was higher in men at 80 years. The mean time elapsed since injury was 7.9 months (±13.3 months). There were 34 patients in group I, 22 patients in group II, and 22 patients in group III.

The clinical characteristics of the study group are shown in Table 1.

No significant differences were found between groups I, II, and III for age, gender, body mass index, urinary tract infections, decubitus ulcers, the AIS grades, as well as thoracic and lumbosacral spine injuries. There were more patients with cervical spine injury in group III (77.3% of patients) compared to group II (40.9%) and group I (32.3%) (*p* = 0.012 for group II vs. III, *p* = 0.001 for group I vs. III). This result is expected because the patients with cervical spine injury often undergo rehabilitation in the chronic phase of SCI due to significant neurologic deficits. Heterotopic ossifications occurred at higher rate in groups II and III (27.3% of patients in each group) compared to group I (2.9%) (*p* = 0.016 for group I vs. II and I vs. III). Low-molecular-weight heparin (LWMH) was used more frequently in group I (94.1% of patients) compared to group II (63.6%) and group III (31.8%) (*p* = 0.006 for group I vs. II, *p* = 0.009 for II vs. III, and *p* = 0.001 for I vs. III). It is clinically justifiable that LMWH is administered most frequently for VTE prophylaxis and treatment in patients with most recent SCI and higher thromboembolic risk.

In the entire study group, seven patients (9% of all patients) were diagnosed with DVT and four patients (5.1%) with superficial vein thrombosis, and all these patients were from group I. No cases of PE were observed in the studied population during hospitalization when the rehabilitation program was conducted. No significant correlations were found between DVT occurrence and the following clinical factors: urinary tract infections, heterotopic ossifications, decubitus ulcers, AIS score, level of spinal injury, or type of paresis. The majority of patients (67.9% of total) received LMWH. No bleeding complications were observed. In addition, we note that patients enrolled in this study had no underlying bleeding disorders. The control group consisted of 42 subjects without SCI including 31 men and 11 women, with an average age of 41.2 years (±15.4 years).

The results of TF, TFPI, and TAT complex assays, which were performed in the study group and in the control group before rehabilitation program, are presented in Table 2. In the study group, the concentration of TF was over threefold higher, the TFPI almost twofold higher, and the TAT complex 4.5-fold higher than in the control group. These differences were statistically significant.

Evaluation of hemostatic parameters in the study group before and after a four-week rehabilitation program is displayed in the Table 3. Before rehabilitation, high TF, TAT complex, and D-dimer concentrations were observed. After rehabilitation, we observed a significant reduction in D-dimer level. TFPI concentrations and AT activity also decreased after the rehabilitation and the differences before and after rehabilitation reached approximately the level of significance. The median values of AT activity and PLT count in SCI patients were within normal limits both before and after rehabilitation.

The results of the measurements of hemostatic parameters in the groups of patients based on time elapsed since SCI are presented in the Table 4 (group I), Table 5 (group II), and Table 6 (group III). Elevated levels of TF, TFPI, and TAT complex, as well as very high D-dimer concentrations were found on admission in patients from group I (3 weeks to 3 months from injury) (Table 4). After four weeks of rehabilitation, in group I we observed a significant reduction in D-dimer levels from a median of 2387 to 1252 ng/mL. There was also a significant reduction in AT activity after rehabilitation in group I, although it stayed within reference ranges both before and after rehabilitation.

Among patients from group II (3 to 6 months after injury) high TF, TFPI, TAT complex, and D-dimer levels were noted on admission (Table 5). A significant decrease in PLT count and TFPI level was observed after rehabilitation in group II.

The patients from group III (>6 months after SCI) demonstrated on admission high concentrations of TF, TFPI, and TAT complex compared to the control group, as well as slightly elevated D-dimer levels (Table 6). A significant increase in TF concentrations was observed after rehabilitation in group III. A decrease in D-dimer levels after rehabilitation was not statistically significant in group III; however, the median value of D-dimer concentration was within normal limits after rehabilitation (Table 6).

The analysis of the measurements of hemostasis parameters in all three groups of SCI patients indicated that D-dimer levels decreased gradually with increasing time elapsed since SCI both at hospital admission (i.e., before rehabilitation) and after rehabilitation, from the highest values in group I before rehabilitation to almost normal levels in group III after rehabilitation (Table 4, Table 5 and Table 6). It should be emphasized that TF, TFPI, and TAT levels were significantly higher in all SCI groups both before and after rehabilitation compared to the control group (Table 2, Table 4, Table 5 and Table 6). However, we did observe a gradual reduction in TF and TAT complex concentrations as time progressed.

## 4. Discussion

To our knowledge, this is the first study to examine the effect of a systematic rehabilitation program on hemostasis in patients with SCI. We found that patients after SCI showed a continued activation of coagulation and fibrinolytic system long term after injury despite participation in a four-week rehabilitation program. Importantly, no decrease in activation of TF-dependent coagulation was observed after rehabilitation, as evidenced by persistently high TF, TFPI, and TAT, regardless of time period within which rehabilitation was carried out. Significant increase in TF level after rehabilitation that was conducted beyond six months after injury suggests that rehabilitation may even induce prothrombotic potential in patients leading a sedentary lifestyle in the chronic phase of SCI. However, D-dimer levels decreased significantly after rehabilitation, the most within 3 months after SCI, which may indicate a reduction of high fibrinolytic potential, especially when rehabilitation was done in the subacute phase of SCI.

Our findings consistently demonstrate the presence of pronounced activation of coagulation in patients hospitalized in both the subacute and chronic phase of SCI, which was manifested by significantly elevated concentrations of TF and to a lesser extent TFPI, in all examined groups of patients regardless of time elapsed since SCI. In addition, we observed that TF level remained elevated after a four-week course of rehabilitation, or even increased significantly in patients undergoing rehabilitation beyond six months after SCI. Moreover, TFPI levels remained high after rehabilitation, although a significant drop in TFPI was observed after rehabilitation that was carried out between three to six months after SCI. Similarly, a high concentration of the TAT complex was observed both before and after rehabilitation in all SCI groups regardless of time elapsed since injury, but particularly in patients up to six months after injury. Beyond six months after SCI, the TAT complex concentrations both before and after rehabilitation were found to be lower, although they still exceeded the values found in the control group more than twofold.

Additionally, we observed a significant contribution of fibrinolysis in the process of hemostasis among patients after SCI. Increased concentrations of D-dimer in SCI patients suggest activation of fibrinolysis, which is secondary to excessive blood coagulation. Activation of fibrinolysis was noted especially in patients who were up to six months after SCI. Fibrinolysis is a natural compensation mechanism in the presence of blood hypercoagulability. Endothelial cells lining the circulatory system produce and secrete tissue plasminogen activator (tPA) into the blood, which degrades micro-clots forming in the endothelium by transforming plasminogen to plasmin [23]. However, our study demonstrated that systematic exercise of moderate intensity led to a reduction of blood D-dimer levels in SCI patients. The greatest reduction of D-dimer levels following rehabilitation was observed in the group of patients undergoing a four-week program of rehabilitation in the subacute phase (up to three months) after SCI, while a smaller effect of rehabilitation was found with increasing time elapsed since injury.

Detailed analysis of study parameters showed varying results depending on the time after SCI when rehabilitation took place. In a group of 34 patients undergoing rehabilitation within the first three months after injury, we observed a decrease of D-dimer level, as well as a reduction in AT activity. In a group of 22 patients who were subject to rehabilitation between three and six months after SCI, exercise led to a decrease of PLT count and TFPI concentration. Rehabilitation that was conducted in 22 patients who were after six months from injury caused a significant increase in TF concentration.

Several publications have reported elevated D-dimer levels in patients after SCI in both the acute and chronic phase [6,24,25]. It should be emphasized that increased D-dimer is often observed in patients after SCI without a diagnosis of VTE [24,25,26]. D-dimer is formed by the degradation of fibrin, the main component of a clot, by plasmin. Boudaoud et al. [24] and Roussi et al. [25] stated that persistently elevated D-dimer levels in the chronic phase after injury were associated with persistent prothrombotic processes and absence of proper fibrinolytic potential. This is consistent with our results, which indicated a persistent activation of coagulation in patients admitted to hospital in the period from three weeks to over six months after injury. The patients after SCI exhibited significantly elevated concentrations of TF, TFPI, and TAT compared to the control group, while increased D-dimer levels, especially within the first three months, indicated persistently increased fibrinolytic potential.

The influence of physical activity on coagulation and fibrinolysis was investigated in various populations and, while some studies reported increased coagulation and fibrinolysis after exertion, others found a reduction [27,28,29,30,31]. In our study, we observed a decrease in D-dimer formation in patients after four weeks of rehabilitation, which was most pronounced in the group of patients who had rehabilitation within the first three months after injury. In addition, the D-dimer levels decreased with time since injury. The decrease in D-dimer after rehabilitation may be related to the rehabilitation itself or to reduced number of complications in the subacute and chronic phase after SCI such as infections, decubitus ulcers, or acute heterotopic ossifications, which may affect activation of coagulation and fibrinolysis [32].

In a study of 4000 elderly men, Wannamethee et al. [27] concluded that physical activity had an inversely proportional effect on many components of hemostasis including PLT count, factors VII and XI, tPA, activated partial thromboplastin time (APTT), D-dimer, as well as inflammatory factors such as C-reactive protein (CRP) and leukocyte count. Their study confirmed that systematic physical activity produced changes in factors involved in coagulation as well as fibrinolysis. In our study, after a four-week rehabilitation program, we observed a reduction in fibrinolysis and persistently high values of the coagulation parameters such as TF and TAT. In contrast, Molz et al. [28] observed an increase in factors involved in fibrinolysis such as D-dimer, plasminogen activator inhibitor (PAI) concentration, and PAI activity immediately after moderate-to-intense exercise as well as during the 30-minute recovery time. These authors suggested that elevated D-dimer levels after exercise and in the first 30 min of recovery indirectly indicate an increase in fibrin formation. They also observed shortening of APTT which could reflect simultaneous activation of coagulation and fibrinolysis during moderate-to-intensive exercise. Other researchers, including van den Burg et al. [33], also observed an increase in D-dimer concentration after physical exercise. Lippi et al. [30] measured D-dimer levels and concentrations of coagulation factors such as VIIa, VIIIa, and von Willebrand Factor (vWF) in professional cyclists and cross-country skiers and compared them to individuals leading sedentary lifestyle, but did not find significant differences between the two groups. Similarly, Clark et al. [31], who evaluated the hemostatic parameters such as D-dimer, tPA, and fibrinogen as well as CRP in volunteers after four weeks of endurance training, did not observe significant changes other than a sudden increase in tPA levels immediately after exercise.

Dimitriadou et al. [34] reported an increased number of PLTs and augmented platelet aggregation in Finish runners after a marathon. Increased PLT counts were also found in sedentary children after moderate exercise, and the elevated PLT levels persisted for 24 h [35]. In our study, however, we did not observe an increased number of PLT after four weeks of rehabilitation but the opposite, i.e., PLT level decreased in group of patients undergoing rehabilitation between three to six months after SCI. Lippi et al. [29] claimed that intense physical activity led to transient hypercoagulability, especially in normally sedentary patients, which might result in thrombotic complications and sudden death. In our study, we observed significant increase in TF concentration after rehabilitation when it was conducted beyond six months after SCI. Such patients are usually wheelchair-bound for many months and their ability to exercise is limited. However, no complications such as DVT were observed in our study in this group of patients.

A review of the literature reveals that changes in hemostasis depend on the intensity of exertion and on when the clotting and fibrinolysis parameters are measured, i.e., whether they are analyzed immediately after single exercise or, as in our study, after a rehabilitation program consisting of several weeks of exercise. Lippi et al. [29] postulated that intense exertion led only to short-lasting, transient changes in coagulation. Van den Burg et al. [33] observed after exercises in healthy subjects with different ages increased concentrations of selected parameters of the intrinsic coagulation pathway in the absence of changes in the extrinsic and common coagulation pathways. They reported increases in concentrations of coagulation factors of the intrinsic pathway and TAT complex, which reflected thrombin formation (common pathway). Further augmentation of components of the intrinsic coagulation pathway occurred immediately after exercise. These authors also demonstrated increased fibrinolysis as reflected by increased D-dimer formation during submaximal and maximal exercise, as well as immediately afterward [33]. Collins et al. [36], on the other hand, examined parameters of hemostasis immediately after physical activity among patients with intermittent claudication vs. healthy subjects. They noted increased TAT and D-dimer levels after exertion in both groups. A review of available studies on the effects of various types of physical activity on hemostasis reveals some disparities with regard to the involvement of extrinsic (TF, TFPI, fVII, PT) or intrinsic (fV, VIII, IX, APTT) coagulation pathways in the processes of hemostasis.

In our study, after four weeks of rehabilitation, we observed no changes in TF (except for elevated levels in patients undergoing rehabilitation during a period that begins more than six months after SCI), TFPI (except for a decrease in patients between three to six months after SCI), and TAT levels. The lack of activation of the extrinsic coagulation pathway after exertion in our study is consistent with results of Weiss et al. [37] and Lund et al. [38], who also observed no increase in TF expression following intensive exercise. Weiss et al. [37] postulated that the extrinsic coagulation pathway was not responsible for the formation of thrombin and fibrin after exercise. Menzel et al. [39], however, studied the influence of maximal and submaximal exertion in a group of young (24 years old) and middle-aged (48 years old) people and observed an increase in the levels of selected components of the extrinsic and intrinsic pathways after exercise.

The incidence of VTE in the general population averages 0.5% among individuals less than 50 years of age and increases with age, reaching 3.8% in patients above the age of 80 [40]. Patients after SCI are usually young and at a very high risk of VTE, especially in the acute phase after injury, as evidenced by the frequency of DVT of 5.4 to 27% and PE of 4 to 5.2% [1,2,3,4,5,6,7]. In our study that included patients in the subacute and chronic phase after SCI, with an average age of 37.7 years, VTE was observed in 9% of patients. The significant disturbances of hemostasis following SCI have been reported to contribute to higher incidence of VTE in the SCI patients [6,9,10,41,42]. Our findings indicate that systematic moderate exercise can affect the hemostasis disorders in patients with SCI and these effects vary depending on time elapsed since injury. The SCI patients participating in an exercised-based rehabilitation program showed a continued activation of coagulation and fibrinolytic system with accompanying depletion of fibrinolysis. In general, these results imply the persisting risk of VTE events in patients participating in the rehabilitation program after SCI. These findings have clinical implications for post-SCI management, especially with regard to the modalities and timing of rehabilitation programs. However, further studies are needed with longer-term follow-up and larger groups of SCI patients, including those at a higher risk of VTE complications in the acute phase of SCI, to identify the prognostic markers for VTE events and address the mechanisms of hemostasis disorders.

## 5. Limitations of the Study

There was no control group that included SCI patients who did not undergo rehabilitation due to the difficulty of accessing such patients, especially in the subacute phase of SCI, up to three months after injury. If patients are not subject to rehabilitation during this period, it is usually because they have severe complications that preclude exercising.

## 6. Conclusions

No decrease in activation of TF-dependent coagulation, as evidenced by persistently high TF, TFPI, and TAT complex levels, was observed in patients with SCI after a four-week rehabilitation program of moderate-intensity exercises regardless of time elapsed since injury. Significant increase in TF levels after rehabilitation that was carried out beyond six-month after SCI implies that exercise may induce additional prothrombotic potential in individuals leading sedentary lifestyle in the chronic phase of SCI. Persistently elevated concentrations of TF, TFPI, and TAT complex were not associated with the occurrence of VTE incidents during the observation period. Significant decrease in D-dimer levels after rehabilitation was observed which may indicate a reduction of high fibrinolytic potential, especially when rehabilitation was carried out in the subacute phase of SCI.

## Figures and Tables

**Table 1:** Study group characteristics: number of patients (*N*) with specific symptoms.

Symptom or Incident	*N (%)*
Tetraplegia	34 (43.6%)
Flaccid paraplegia	18 (23.1%)
Spastic paraplegia	26 (33.3%)
Cervical spine injury	37 (44.4%)
Thoracic spine injury	33 (42.3%)
Lumbosacral spine injury	8 (10.3%)
AIS A	34 (43.6%)
AIS B	30 (36.5%)
AIS C	14 (17.9%)
Decubitus ulcers	11 (14.1%)
Urinary tract infection	26 (33.3%)
Heterotopic ossifications	13 (16.6%)
Administration of LMWH	53 (67.9%)
Deep vein thrombosis	7 (9.0%)
Superficial vein thrombosis of the lower limbs	4 (5.1%)
Pulmonary embolism	0

Data represent the number of patients (*N*) including the percentage of total number (%). Abbreviations: AIS—American Spinal Injury Association Impairment Scale; AIS A—impairment is complete according to AIS; AIS B and C—impairment is incomplete according to AIS; LMWH—low-molecular-weight heparin.

**Table 2:** Plasma concentrations of TF, TFPI, and TAT complex in the study group vs. the control group before four-week rehabilitation program.

	Study Group *N* = 78		Control Group *N* = 42		*p*-Value between Groups
Parameter	Median	Q25/Q75	Median	Q25/Q75	
TF (pg/mL)	453.84	327.30/567.66	128.26	90.44/183.40	0.000
TFPI (ng/mL)	103.26	59.36/131.50	59.90	43.30/96.76	0.001
TAT (ng/mL)	8.75	5.29/17.54	2.20	1.98/3.36	0.000

Abbreviations: TAT—thrombin–antithrombin complex; TF—tissue factor; TFPI—tissue factor pathway inhibitor; Q25—lower quartile; Q75—upper quartile.

**Table 3:** Parameters of hemostasis in the study subjects before and after a four-week rehabilitation program.

Test (*N* = 78)		Before Rehabilitation		After Rehabilitation		*p*-Value between Groups
Parameter	Units	Median	Q25/Q75	Median	Q25/Q75	
PLT count	g/L	252.00	204.00/352.00	249.00	211.00/317.00	0.116
TF	pg/mL	453.84	327.30/567.66	459.76	348.20/546.84	0.371
TFPI	ng/mL	103.26	59.36/131.50	92.34	52.84/132.04	0.075
AT	%	101.95	94.80/112.00	101.05	91.40/106.70	0.071
TAT	ng/mL	8.75	5.29/17.54	6.70	4.50/10.88	0.48
D-dimer	ng/mL	1249.50	600.00/2540.00	903.50	430.00/1743/00	0.001

Abbreviations: AT—antithrombin activity; PLT—platelet; TAT—thrombin–antithrombin complex; TF—tissue factor; TFPI—tissue factor pathway inhibitor; Q25—lower quartile; Q75—upper quartile.

**Table 4:** Parameters of hemostasis before and after a four-week rehabilitation program in patients from group I (three weeks to three months after injury).

Test (*N* = 34)		Before Rehabilitation		After Rehabilitation		*p*-Value between Groups
Parameter	Units	Median	Q25/Q75	Median	Q25/Q75	
PLT count	g/L	262.0	242.0/352.0	255.0	218.0/348.0	0.180
TF	pg/mL	482.1	338.9/572.6	492.2	366.6/582.4	0.447
TFPI	ng/mL	90.5	52.8/155.9	93.3	47.7/139.9	0.301
AT	%	102.5	97.4/114.9	100.2	91.1/105.0	0.009
TAT	ng/mL	10.3	5.9/18.9	7.5	5.8/12.6	0.326
D-dimer	ng/mL	2387.5	1250.0/5190.0	1252.0	580.0/2240.0	0.000

Abbreviations: AT—antithrombin activity; TAT—thrombin–antithrombin complex; TF—tissue factor; TFPI—tissue factor pathway inhibitor; Q25—lower quartile; Q75—upper quartile.

**Table 5:** Parameters of hemostasis before and after a four-week rehabilitation program in patients from group II (three months to six months after injury).

Test (*N* = 22)		Before Rehabilitation		After Rehabilitation		*p*-Value between Groups
Parameter	Units	Median	Q25/Q75	Median	Q25/Q75	
PLT count	g/L	322.00	229.00/363.00	262.50	216.00/322.00	0.042
TF	pg/mL	453.84	337.59/617.60	451.31	333.12/506.50	0.485
TFPI	ng/mL	111.34	72.20/143.44	88.32	60.96/139.70	0.022
AT	%	97.65	90.30/111.00	99.10	87.00/107.80	0.126
TAT	ng/mL	10.40	7.18/23.48	9.66	4.50/15.22	0.906
D-dimer	ng/mL	1033.50	515.00/1800.00	935.50	430.00/1475.00	0.733

Abbreviations: AT—antithrombin activity; TAT—thrombin–antithrombin complex; TF—tissue factor; TFPI—tissue factor pathway inhibitor; Q25—lower quartile; Q75—upper quartile.

**Table 6:** Parameters of hemostasis before and after a four-week rehabilitation program in patients from group III (more than six months after injury).

Test (*N* = 22)		Before Rehabilitation		After Rehabilitation		*p*-Value between Groups
Parameter	Units	Median	Q25/Q75	Median	Q25/Q75	
PLT count	g/L	205.50	181.00/265.00	221.00	204.00/262.00	0.372
TF	ng/mL	388.90	290.25/532.81	442.09	357.50/498.14	0.050
TFPI	ng/mL	93.75	59.36/118.12	104.46	58.36/126.60	0.808
AT	%	102.55	94.00/109.20	104.05	100.50/112.00	0.095
TAT	ng/mL	5.40	3.65/11.52	5.15	3.06/6.26	0.091
D-dimer	ng/mL	522.00	284.00/1057.00	420.00	262.00/711.00	0.768

Abbreviations: AT—antithrombin activity; TAT—thrombin–antithrombin complex; TF—tissue factor; TFPI—tissue factor pathway inhibitor; Q25—lower quartile; Q75—upper quartile.
